# Phase-Selective Synthesis of CIGS Nanoparticles with Metastable Phases Through Tuning Solvent Composition

**DOI:** 10.1186/s11671-018-2781-1

**Published:** 2018-11-14

**Authors:** Xiaokun Zhang, Shuai Liu, Fang Wu, Xiaoli Peng, Baoguo Yang, Yong Xiang

**Affiliations:** 1 School of Materials and Energy, University of Electronic Science and Technology of China, 2006 Xiyuan Avenue, West High-Tech Zone, Chengdu, 611731 Sichuan China; 2 Science and Technology on Electronic Test and Measurement Laboratory, The 41st Research Institute of CETC, Qingdao, 266555 Shandong China

**Keywords:** CIGS, Nanoparticles, Metastable, Wurtzite, Zincblende, Phase-selective

## Abstract

I-III-VI_2_ compounds have shown great interests in the application of functional semiconductors. Among them, Cu(In,Ga)S_2_ has been a promising candidate due to its excellent optoelectronic properties. Although the polymorphs of Cu(In,Ga)S_2_ have been attracted extensive attentions, the efforts to developing the methodologies for phase-controlled synthesis of them are rare. In this paper, we reported a phase-selective synthesis of CIGS nanoparticles with metastable phases via simply changing the composition of solvents. For the wet chemistry synthesis, the microstructure of the initial nuclei is decisive to the crystal structure of final products. In the formation of Cu(In,Ga)S_2_, the solvent environment is the key factor, which could affect the coordination of monomers and influence the thermodynamic conditions of Cu-S nucleation. Moreover, wurtzite and zincblende Cu(In,Ga)S_2_ nanoparticles are selectively prepared by choosing pure en or its mixture with deionized water as reaction solvent. The as-synthesized wurtzite Cu(In,Ga)S_2_ possess a band gap of 1.6 eV and a carrier mobility of 4.85 cm^2^/Vs, which indicates its potential to construct a heterojunction with hexagonal-structured CdS for solar cells.

**Electronic supplementary material:**

The online version of this article (10.1186/s11671-018-2781-1) contains supplementary material, which is available to authorized users.

## Introduction

I-III-VI_2_ compound semiconductors hold great promises for the applications of photoelectric devices, due to their advantageous optical and electrical properties [1–5]. Especially, the thin-film solar cells based on Cu(In,Ga)Se_2_ (CIGSe) have achieved the energy conversion efficiency higher than 22% [6]. However, compared with silicon-based photovoltaic technologies, the cost of solar cells based on compound semiconductors still need to be reduced significantly [7]. Recently, CuInS_2_ (CIS) and Cu(In,Ga)S_2_ (CIGS), in which selenium (Se) is replaced by the less toxic and cheaper sulfur (S), garnered great interests as appealing alternatives to CIGSe [8–10]. Besides the choice of materials, it is generally believed that the non-vacuum process based on the suspension of nanoparticles (NPs) is also promising in the reduction of the cost of CIGS-based solar cells [7, 11]. Therefore, CIGS NPs play a vital role in the development of low-cost thin-film solar cells [12–14].

In the past two decades, many efforts have been made to synthesize the chalcogenide NPs with desired properties for photovoltaic application [15–21]. Phase control is one of the most important point for the synthesis of chalcogenide NPs because their optoelectronic properties depend on the crystal structure [22–26]. According to literatures [27–29], CIGS may have three polymorphs: a thermodynamically stable phase with tetragonal chalcopyrite (CH) structure and two metastable phases with cubic zincblende (ZB) structure and hexagonal wurtzite (WZ) structure, respectively. Previous studies mainly focused on the CH-structured CIS and CIGS [18, 30, 31]. Metastable ZB- and WZ-structured CIS NPs prepared via hot-injection approach were firstly reported by Pan et al. in 2008 [32]. After that, CIS NPs with metastable phase were also obtained by solvothermal synthesis [33, 34] and thermal decomposition of precursors [35, 36]. However, to our best knowledge, all these previous reports on the synthesis of metastable phase CIS or CIGS invlove inducible additives [37, 38], expensive ligands [32, 39], or complex precursors [34–36]; a low-cost and facile synthesis of CIGS NPs still remains challenging.

The formation of NPs generally includes two steps, namely the nucleation step and the crystalline growth step [40, 41]. Previous studies [26, 42, 43] consistently indicate that CIGS NPs were formed in two steps: (1) the formation of Cu-S crystal nuclei via the rapid reaction between Cu^+^ and S^2−^; and (2) the incorporation of indium and gallium into the Cu-S crystal nuclei through the diffusion and/or cation exchanging. In addition, the crystal structure of final CIGS NPs may mainly depend on the structure of Cu-S nuclei [25, 35, 44]. Herein, we report a one-pot solvothermal synthesis of CIGS NPs with metastable phases, without the requirements of additives, expensive ligands, or preparation of complex precursors. By simply changing the composition of solvents for solvothermal reaction, the thermodynamic environment of Cu-S nucleation can be tuned, in turn ZB- or WZ-structured CIGS NPs can be selectively obtained. In addition to the discussion of the mechanism of phase-selective synthesis, the electrical and optical properties of the products with different crystal structures are also compared in this study.

## Experimental Methods

### Materials

CuCl_2_·2H_2_O (99.9%), sulfur powder (99.9%), anhydrous ethylenediamine (en, 99%), and anhydrous ethanol (99.7%) were purchased from Chengdu Kelong Chemical Co., Ltd. InCl_3_ (99.9%) was purchased from Aladdin. Ga(acac)_3_ (99.99%) was purchased from Sigma-Aldrich. All the chemicals were used as received.

### Solvothermal Synthesis of CIGS NPs

For the synthesis of WZ-structured CIGS NPs, a metal precursor solution was prepared by dissolving CuCl_2_·2H_2_O (0.164 g, 0.96 mmol), InCl_3_ (0.192 g, 0.868 mmol), and Ga(acac)_3_ (0.068 g, 0.186 mmol) in 5 ml ethylenediamine. Sulfur powder (0.0826 g, 2.58 mmol) was dispersed in 5 ml ethylenediamine through an ultrasonic treatment at 60 °C for 15 min. The metal precursor solution was mixed with the dispersion of sulfur in a 20-ml Teflon-lined autoclave. About 6 ml ethylenediamine was then added to make the volume of the reaction solution is 80% of that of the Teflon-lined autoclave. The autoclave was sealed and treated with sonication at 60 °C for 15 min. Thereafter, the autoclave was put into an oven. The temperature in the oven was raised from room temperature to 200 °C and maintained for 24 h, and then cooled down to room temperature naturally. The precipitate was separated by centrifugation, washed with a mixed solvent of ethanol and deionized water for five times, and dried in vacuum at 60 °C for 4 h.

For the synthesis of ZB-structured CIGS NPs, the metal precursor solution was prepared by dissolving metal salts in 5 ml deionized water. Other procedures and synthetic conditions were unchanged.

### Characterization

The phase of the as-synthesized NPs was identified by X-ray diffraction (XRD) on a Bruker D8 Advance diffractometer equipped with monochromatized Cu-Kα (λ = 1.5418 Å) radiation. The diffraction data was collected with an angle increment of 0.02° at a scan rate of 0.1 s/step. Scanning electron microscopy (SEM) images were obtained using a ZEISS EVO^@^ LS15 SEM operated under 15 kV. The ZEISS EVO^@^ LS15 SEM is equipped with a Bruker Nano GmbH XFlash Detector 5010, which was used to estimate the stoichiometric proportion by energy dispersive X-ray spectroscopy (EDS). The optical properties of the as-prepared products were characterized by an Agilent Cary5000 UV-Vis-IR spectrophotometer. The electrical properties were calculated based on Hall effect measurements using Swin Hall 8800 system. The as-synthesized CIGS NPs were deposited on glass substrates via spray coating, and electrical contacts were formed by silver paste for Hall effect measurements. The species in precursor solutions were studied by UV-Vis-IR spectrophotometer (Agilent Cary5000) and Raman spectrophotometer (Renishaw Invia).

## Results and Discussion

**Fig. 1:**
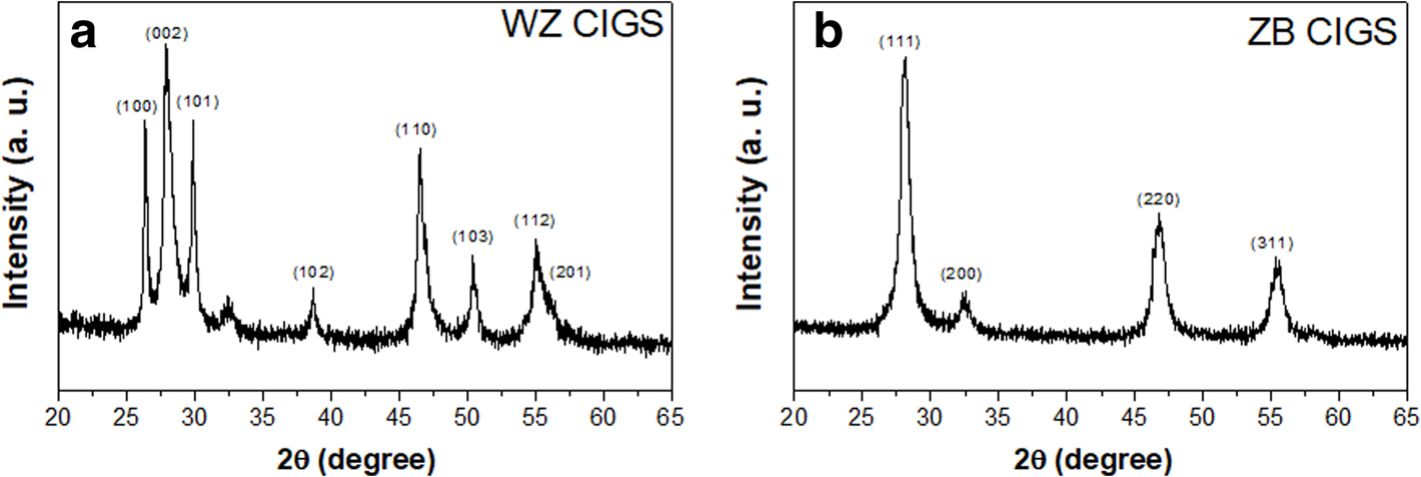
XRD patterns of CIGS NPs synthesized in pure en solvent (**a**) and en/water mixture solvent (**b**)

For the synthesis of CIGS NPs, sulfur (S) dispersed in ethylenediamine (en) was mixed with the metal salts that dissolved in en or deionized water. And then, the mixtures with different solvent compositions were ultrasonic treated at 60 °C for 15 min, followed by reacted at 200 °C for 24 h under the solvothermal condition. en with a dual amine group and a short carbon chain was used to stabilize the metastable phase CIGS. As shown in Fig. 1, the XRD peaks of the as-synthesized NPs are well identical with the reported wurtzite CIS pattern [32–34], indicating that crystal of the fabricated NPs derived from pure en solvent is a hexagonal wurtzite structure. Meanwhile, for NPs prepared in the mixing solvent of en and deionized water, the XRD pattern is well consistent with that of the ZB-structured CIS [32, 34, 39]. Thus, the phase-selective synthesis of CIGS NPs with metastable phase can be achieved by simply altering the composition of reaction solvent.

**Fig. 2:**
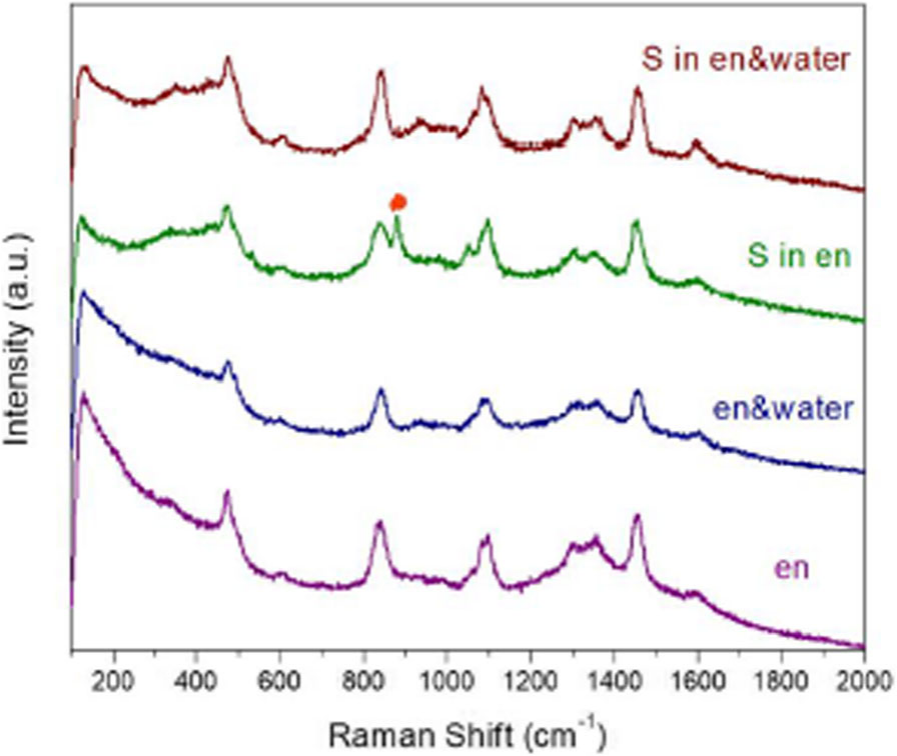
Raman spectra of pure en (purple line), the mixture of en and deionized water (blue line), the solutions of the S precursor in pure en (green line), and the solutions of the S precursor in the mixture of en and deionized water (red line)

**Fig. 3:**
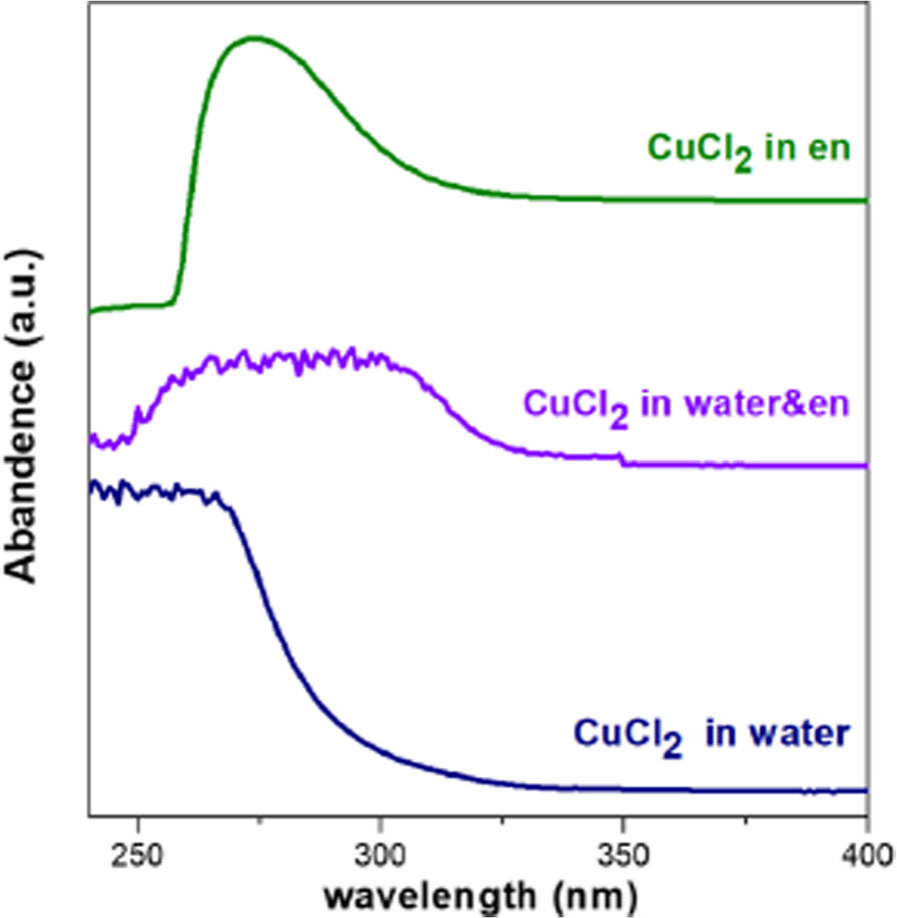
Absorption spectra of the solutions of the Cu precursor in en (green line), deionized water (blue line), and the mixture of en and deionized water (violet line)

As mentioned above, the nucleation of Cu-S clusters is kinetically favorable and decisive for the crystal structure of final quaternary products. The solvent environment may affect the reactivity of the metal salt and chalcogen precursors, in turn influence the microstructure of Cu-S clusters. It is known that en is a good attacking agent and can partially reduce the chalcogen precursor [35, 45]. To investigate the impact of solvent composition on the status of sulfur, the Raman spectra of en solvent, en/water mixture, the solution of S in en, and the solution of S in en/water mixture were collected. As shown in Fig. 2, a split peak at 811 cm^−1^ can be observed in the spectrum of the solution of S in en, while it is absent from the other three counterparts. This split peak may indicate the status of sulfur precursor in the pure en is different from that in the en/water mixture. It seems that the introduction of H_2_O would weaken the interaction between S and amino group (see the blue line in Fig. 2). Furthermore, en can act as a strong chelating agent for metal ions due to its feature of dual amine group. The amine coordination to Cu^2+^ differs in the pure en and the mixture of en and deionized water, which is indicated by the fact that the color of the en solution of CuCl_2_ is dark green while that of aqueous solution of CuCl_2_ is blue. Based on the stability constant and dissociation equilibrium of complexes, the molar concentration of free Cu^2+^ in the pure en and water are evaluated to be 3.12 × 10^−22^ M and 0.192 M, respectively (see the calculation details in Additional file 1). Raman spectra of the solutions of CuCl_2_ in pure en and the mixture of en and water seem to be similar (Additional file 1: Figure S1). This should ascribe to the vibrations of Cu–NH_2_ chelating bond exists in both two solutions. Figure 3 shows absorption spectra of the solutions of CuCl_2_ in pure en, water, and their mixture. The broad absorption at 250–350 nm of CuCl_2_ in the mixture solvent implies that the coordinating status of Cu^2+^ may be a balanced combination of that in pure en and water. Take the evaluated concentration of free Cu^2+^ in consideration, it is reasonable to propose that there are a larger number of free monomers ready to react with S precursor at a relative low temperature in the en/H_2_O mixture than in that pure en. For the reaction in the mixture solvent, the free Cu^2+^ may react with elemental S to from Cu-S nuclei at moderate temperature. Meanwhile, the nucleation of Cu-S in pure en may happen at elevated temperature between Cu^2+^ and ${S}_n^{2-}$, since high temperature facilitates the dissociation of Cu[en]^2+^ complex and the reduction of elemental S by pure en [45]. Thus, the different solvent environments result in the different thermodynamic conditions and reacting species for Cu-S nucleation, in turn leading to different microstructures of Cu-S clusters. After incorporation of In and Ga into the Cu-S clusters, CIGS NPs with different crystal structure can be obtained from pure en and its mixture with water, respectively. According to the mechanism presented here, the minor peak at ~ 32° in Fig. 1a, which may be indexed to ZB-structured CIGS, should attribute to the trace water existing in en solvents.

**Fig. 4:**
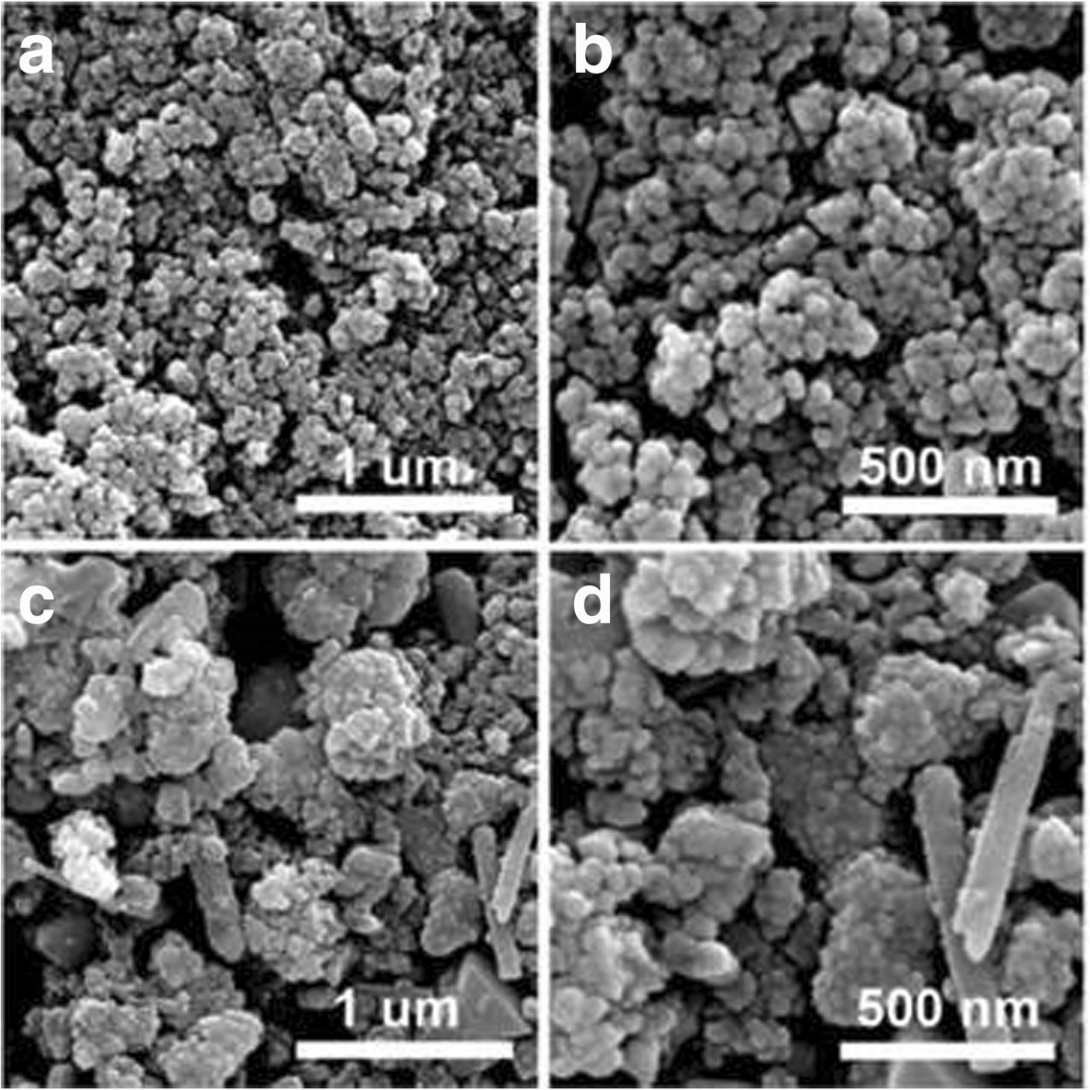
Representative SEM images of WZ-structured (**a**, **b**, **c**) and ZB-structured (**c**, **b**, **d**) CIGS NPs

The composition of reaction solvents can also affect the morphologies of as-prepared CIGS NPs. The WZ-structured CIGS NPs, which is derived from pure en, exhibit a uniform particulate morphology with a diameter of 50 nm and are fairly monodispersed (Fig. 4a, b). However, the monodispersity of ZB-structured CIGS NPs is poor, and its morphology is much more complex. The nanoscale pellets, flakes, and rods can be observed in Fig. 4c, d. Such a morphology difference is consistent with the solvent-dependent reaction mechanism proposed above. For the synthesis of WZ-structured CIGS NPs in pure en, the nucleation is difficult at a low temperature because of the chelating bond between en and Cu^2+^. At an elevated temperature, free metallic monomers are provided by the dissociation of complex compounds, and S precursor is in a reactive and soluble form of ${S}_n^{2-}$. High concentration of monomers and homogeneous reaction environment trend to generate numerous Cu-S nuclei. Thus, most of monomers are consumed by the nucleation, and the growth of the clusters is limited. This process is beneficial to the uniform and fine morphology of the resultant NPs. Meanwhile, the nucleation of Cu-S is possible to happen at a low temperature during the synthesis of ZB-structured CIGS, because considerable free Cu^2+^ exist in the mixture of en and water at a room temperature, and they may react with solid elemental S. The supplementary reactive monomers provided at high temperature would trend to facilitate the growth of clusters but not to generate new nuclei, through reaction at the solution-solid interfaces [40, 41]. In addition, en molecular cannot provide a strong spatial confinement effect to the clusters during its growth, because the segments of en molecular are short. Thus, ZB-structured CIGS NPs show various shapes, such as pellets, flakes, and rods. Slight clustering of the NPs observed in SEM images may be caused by the solvent evaporation during testing sample preparation.

The composition of the as-prepared CIGS NPs was determined using EDS, and the results were summarized in Table 1. The element composition in WZ- and ZB-structured CIGS NPs is approximately consistent with the stoichiometric chemical composition. However, both of them show a slight sulfur-poor feature, i.e., [0.5Cu+1.5(In+Ga)]:S = 1:0.96 and 1:0.91 for WZ- and ZB-structured GIGS respectively, even though ~ 35% excess sulfur was applied in the starting materials. This deviation from stoichiometry can be remedied by a heat treatment in sulfur atmosphere during the fabrication of CIGS absorber layers via NPs-based non-vacuum approaches. It can be seen that WZ-structured CIGS NPs possess a copper-poor characteristic (Cu:(In+Ga) = 1:1.16) which is desirable for photovoltaic applications [46], while the ZB-structured one is slight copper-rich (Cu:(In+Ga) = 1:0.96). This indicates that the solvent composition may have a kinetic influence on the incorporation of In and Ga into Cu-S clusters. The pure en environment might induced a favorable kinetics for In and Ga incorporation.

**Table 1:** Composition of WZ- and ZB-structured CIGS NPs (calculated by the measured atomic ratios derived from EDS, the listed ratios are the average of 6 measured values from three individual samples)

Sample	Cu:In:Ga:S	Cu:(In+Ga)	[0.5Cu+1.5(In+Ga)]:S
WZ-CIGS	1:0.86:0.30:2.16	1:1.16	1:0.96
ZB-CIGS	1:0.68:0.28:1.76	1:0.96	1:0.91

The optical and electrical properties of as-synthesized CIGS NPs with different crystal structures were investigated and compared. The band gap energy (E_g_) of WZ- and ZB-structured CIGS NPs was estimated based on the UV-vis-IR absorption spectra [47]. Both of them exhibit *E*_g_ around 1.6 eV (Additional file 1: Figure S2), which is attractive for the photovoltaic devices [48]. According to the Hall effect measurement, all the synthesized CIGS NPs show a N-type conducting behavior, which should attribute to their sulfur-poor feature. The values of sheet resistivity, carrier concentration, and mobility listed in Table 2 are comparable to the reported values [49, 50]. These results further suggest that the as-synthesized WZ-structured CIGS possess superior electrical properties over ZB-structured one. It is reported that a sulfur-rich interface between CuInSe_2_ and CdS would improve the efficiency of solar cells because of the optimized bandgap structure [51]. The outstanding optical and electrical properties make WZ-CIGS NPs very attractive for the construction of heterojunctions with WZ-CdS. Besides the bandgap optimization, the WZ-CIGS/WZ-CdS heterojunctions may also benefit from better lattice-matching because both of them are hexagonal-structured. It is worth to note that the measured carrier mobility of WZ-CIGS is as high as 4.85 cm^2^/Vs, which is comparable to that of indium gallium zinc oxide (IGZO) (~ 5–10 cm^2^/Vs) [52]. It is generally believed that IGZO is one of the most promising candidates for next-generation display panel [53]. Thus, we believe CIGS also hold a great promise for optoelectronic applications.

**Table 2:** Electrical properties calculated from the Hall effect measurement

Sample	Conduction type	Sheet resistivity (Ω/□)	Carrier concentration (cm^−3^)	Mobility (cm^2^/Vs)
WZ-CIGS	N-type	9.9 × 10^5^	8.53 × 10^14^	4.85
ZB-CIGS	N-type	4.1 × 10^7^	4.27 × 10^14^	0.34

To verify the feasibility of the presented strategy for the phase-selective synthesis of copper-based multinary chalcogenides, CIS and CuGaS_2_ (CGS) were also prepared using pure en or the mixture of en and water as solvents. XRD patterns of the products demonstrate that the CIS and CGS with WZ and ZB crystal structures were selectively synthesized (Additional file 1: Figure S3). The successfully synthesis of CIS, CGS, and CIGS with phase-selectivity indicates that the presented approach also possess the ability to tune the In/Ga ratio of products easily, in turn the *E*_g_ of the compound semiconductors can be engineered, which is valuable for the preparation of absorber materials for solar cells [54].

## Conclusion

In summary, phase-selective synthesis of CIGS NPs with metastable phases is achieved by simply changing the composition of reaction solvents. The amine coordination to metallic monomers differs in pure en and the mixture of en and deionized water. And thus, the thermodynamic condition of nucleation of Cu-S, which is kinetically preferred during the solvothermal synthesis, can be influenced by solvent environment. WZ- and ZB-structured CIGS NPs are selectively prepared through controlling the microstructure of pre-formed Cu-S nuclei. The resultant WZ-structured CIGS NPs exhibit a uniform morphology and excellent optical and electrical properties. In addition to providing an alternative approach for the synthesis of high-quality CIGS NPs in a phase-controlled manner, the strategy presented in this study may also contribute to developing methodologies for phase-selective synthesis of other polymorph systems.

## Additional file

Additional file 1: Calculation of the concentration of free Cu^2+^. **Figure S1.** Raman spectra of the solutions of the Cu precursor in ethylenediamine (blue line), and the mixture of ethylenediamine and deionized water (green line). **Figure S2.** The band gap energy of WZ-(blue) and ZB-structured (pink) CIGS NPs. **Figure S3.** XRD patterns of CIS (a, b) and CGS (c, d) NPs with WZ (a, c) and ZB (b, d) crystal structures. (DOCX 405 kb)

## Abbreviations

CIGS: Cu(In,Ga)S_2_
CIGSe: Cu(In,Ga)Se2
CIS: CuInS2
en: Ethylenediamine
NPs: Nanoparticles
WZ: Wurtzite
ZB: Zincblende
