# Supplementary material for: Phase-Selective Synthesis of CIGS Nanoparticles with Metastable Phases Through Tuning Solvent Composition
Source: Nanoscale Res Lett. 2018 Nov 14;13:362. doi: 10.1186/s11671-018-2781-1 (PMC6235770; doi:10.1186/s11671-018-2781-1)
Supplement: Supplementary file 1 — Calculation of the concentration of free Cu2+. Figure S1. Raman spectra of the solutions of the Cu precursor in ethylenediamine (blue line), and the mixture of ethylenediamine and deionized water (green line). Figure S2. The band gap energy of WZ-(blue) and ZB-structured (pink) CIGS NPs. Figure S3. XRD patterns of CIS (a, b) and CGS (c, d) NPs with WZ (a, c) and ZB (b, d) crystal structures. (DOCX 405 kb) [file 11671_2018_2781_MOESM1_ESM.docx]

**Additional file for**

**Phase-selective synthesis of CIGS nanoparticles with metastable phases through tuning solvent composition**

*Xiaokun Zhang,^1^ Shuai Liu,^1^ Fang Wu,^1^ Xiaoli Peng,^1^ Baoguo Yang,^2^ Yong Xiang^1*^*

1 School of Materials and Energy, University of Electronic Science and Technology of China, 2006 Xiyuan Avenue, West High-Tech Zone, Chengdu, Sichuan 611731, China

2 Science and Technology on Electronic Test & Measurement Laboratory, The 41st Research Institute of CETC, Qingdao, Shandong 266555, China

* Correspondence: xyg@uestc.edu.cn

1. **Calculation of the concentration of free Cu^2+^**

In the solutions of CuCl_2_ in ethylenediamine (en) or H_2_O, Cu^2+^ may be in the form of [Cu(en)]^2+^ or [Cu(H_2_O)]^2+^, respectively. The dissociation balance of these complexes could be described by equation S1 and S2.

Cu^2+^+en ⇋ [Cu(en)]^2+^ (S1)

Cu^2+^+H_2_O ⇋ [Cu(H_2_O)]^2+^ (S2)

The stability constant of complexes (*K_f_*) is defined as

$\text{K}_{\text{f}}\text{ }\text{=}\text{ }\frac{\text{[}\text{M}\text{L}_{\text{n}}\text{]}}{\text{[}\text{M}^{\text{x+}}\text{]}{\text{[}\text{L}\text{]}}^{\text{n}}}$ (S3)

where $\text{[}\text{M}\text{L}_{\text{n}}\text{]}$, $\text{[}\text{M}^{\text{x+}}\text{]}$, and $\text{[}\text{L}\text{]}$ are the molar concentration of complexes, free metallic ions, and ligands, respectively. n is the coordinating number. Since H_2_O is used as cardinality for calibrating the *K_f_* of other solvents, the molar concentration of free metallic ions in the H_2_O is assumed to be equal to the molarity of metal salts.

For this study, 0.96 mmol CuCl_2_ is dissolved in 5 ml en and H_2_O, respectively. The concentration of free Cu^2+^ in water solution is

$\left[ {Cu}^{2+} \right]=\frac{0.96\times{10}^{-3}}{5\times{10}^{-3}}M=0.192 M$ (S4)

The stability constant of [Cu(en)]^2+^ is

$\text{K}_{\text{f,}\text{Cu(en)}_{\text{2}}^{\text{2+ }}}\text{= 4×}\text{10}^{\text{19}}$ (S5)

The concentration of free Cu^2+^ in en solution is

[Cu^2+^]_en_=3.12×10^-22^ M (S6)

1. **Supplementary characterizations**


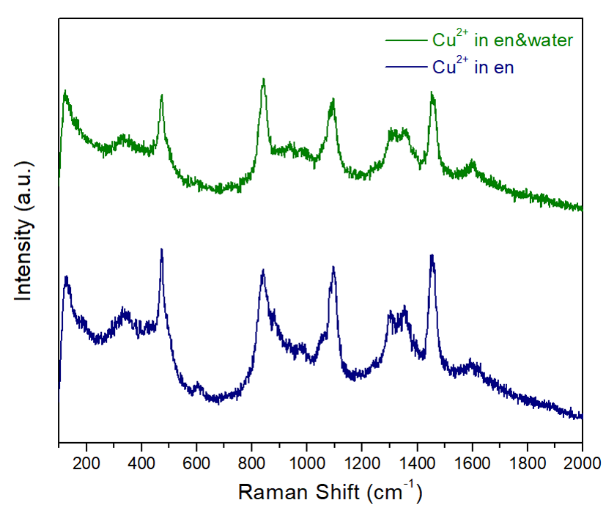


Figure S1. Raman spectra of the solutions of the Cu precursor in ethylenediamine (blue line), and the mixture of ethylenediamine and deionized water (green line)


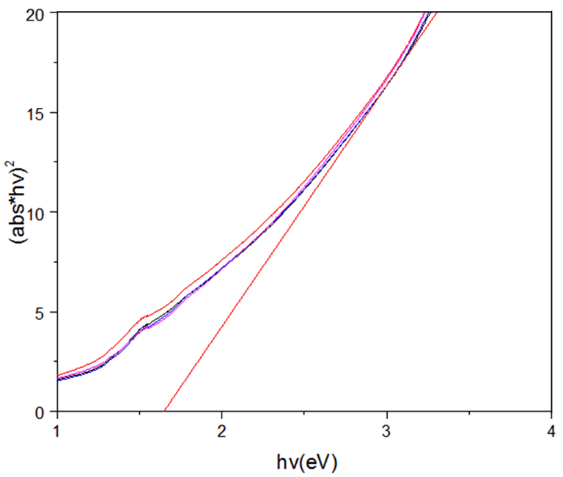


Figure S2. the band gap energy of WZ-(blue) and ZB-structured(pink) CIGS NPs


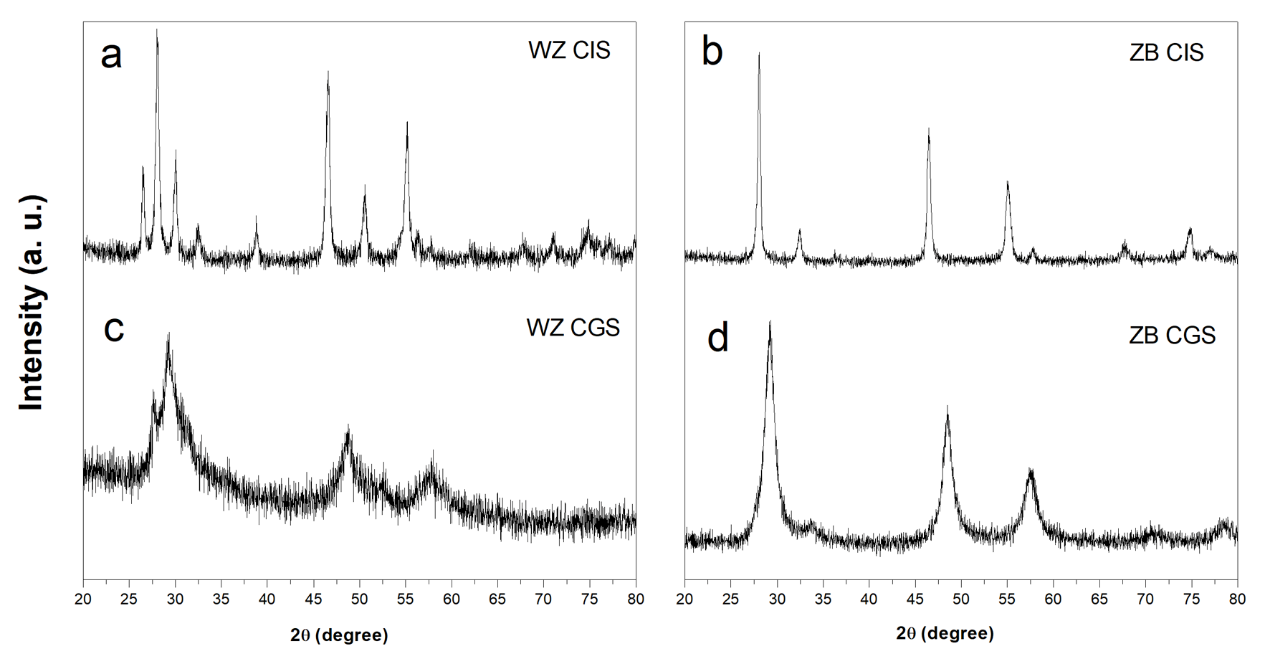


Figure S3. XRD patterns of CIS (a, b) and CGS (c, d) NPs with WZ (a, c) and ZB (b, d) crystal structures
